# Delivery of Health Care by Spanish Dental Hygienists in Private and Public Dental Services during the COVID-19 De-Escalation Phase (June 2020): A Cross-Sectional Study

**DOI:** 10.3390/ijerph18168298

**Published:** 2021-08-05

**Authors:** Antonio Javier Expósito-Delgado, Verónica Ausina-Márquez, María Victoria Mateos-Moreno, Elena Martínez-Sanz, María del Carmen Trullols-Casas, María Eulalia Llamas-Ortuño, José María Blanco-González, Teresa Almerich-Torres, Manuel Bravo, Yolanda Martínez-Beneyto

**Affiliations:** 1 Servicio Andaluz de Salud, National Health Service, 23200 Jaén, Spain; antonioj.exposito.sspa@juntadeandalucia.es; 2 Department of Paediatric and Preventive Dentistry, Faculty of Dentistry, European University of Valencia, 46001 Valencia, Spain; veronica.ausina@universidadeuropea.es; 3 Department of Dental Clinical Specialties, Faculty of Dentistry, University Complutense of Madrid, 28040 Madrid, Spain; 4 Department of Anatomy and Embryology, Faculty of Medicine, University Complutense of Madrid, 28040 Madrid, Spain; emartinez-sanz@med.ucm.es; 5 Servei Balear de Salut, National Health Service, 07005 Palma de Mallorca, Spain; mctrullols@gmail.com; 6 Servicio de Salud de Castilla la Mancha, National Health Service, 13700 Tomelloso, Spain; eulaliallamas@gmail.com; 7 Dirección General de Salud Pública, Servicio de Salud del Principado de Asturias, National Health Service, 33005 Oviedo, Spain; josemaria.blancogonzalez@asturias.org; 8 Department of Preventive Dentistry, Faculty of Medicine-Dentistry, University of Valencia, 46001 Valencia, Spain; teresa.almerich@uv.es; 9 Department of Preventive & Community Dentistry, Faculty of Dentistry, University of Granada, 18001 Granada, Spain; mbravo@ugr.es; 10 Department of Preventive & Community Dentistry, Faculty of Medicine-Dentistry, University of Murcia, 30001 Murcia, Spain; yolandam@um.es

**Keywords:** COVID-19, SARS-CoV-2, health personnel, dental hygienists, surveys and questionnaires, dental care, infection control, personal protective equipment

## Abstract

Background: The first wave of the COVID-19 pandemic in Spain posed a major challenge for Spanish dental professionals. The objective of this work is to describe the dental hygienists’ work status and employment patterns during the de-escalation phase in order to analyse the standards of knowledge, compliance with official recommendations, and dental activities both in the public health service and in the private sector. Material and Methods: A cross-sectional questionnaire was answered by Spanish dental hygienists via WhatsApp, Facebook, and Instagram. The questionnaire was piloted before it was distributed and carried out during June 2020. Results: Here, 517 dental hygienists were surveyed, of which 86.2% followed the official recommendations to avoid contagion and 63.8% agreed with the gradual return to work by limiting the use of aerosols. Private dental hygienists identified more with returning to work without restrictions (14.5%) versus those working for the public service (1.2%) (*p* < 0.005). Conclusions: Dental hygienists’ return to work has involved different strategies, aimed at controlling infection and guaranteeing the safety of patients and the rest of the dental team. The availability of personal protective equipment, the adaptation of clinical infrastructure, and patient care management have differed between professionals working in the private and public sectors.

## 1. Introduction

The SARS-CoV-2 pandemic emerged in Spain in mid-February 2020 through isolated cases and began to exponentially spread [1]. The level of spread, the severity, and the alarming lack of inaction prompted the World Health Organization (WHO) to declare the coronavirus outbreak a global pandemic [2]. On 14 March 2020, the Government of Spain decreed a state of alarm in order to deal with the expansion of the coronavirus [3].

The supply chain of personal protective equipment (PPE) collapsed during the first weeks after the declaration of the pandemic. This was due to the global panic that multiplied the speculation and demand for these types of products [4]. The direct consequence was that health professionals working on the frontlines found themselves unprotected due to a lack of the PPE needed to cope with the COVID-19 disease [4,5,6].

The risk of infection with SARS-CoV-2 has not been the same in all professions. Among their activities, a dental hygienist’s role involves the elimination of calculus, staining, scaling, polishing, or non-invasive preventive treatments [7]. Most of these dental procedures generate tiny liquid particles, called ‘aerosols’, which float in the air [8,9]. There is growing evidence indicating that the virus can be transmitted remotely through aerosols and in samples of exhaled breath [8,10,11]; therefore, in dental consultations, the virus can be transmitted through the air, through droplets generated in treatment procedures, and by person-to-person contact [9,10]. Despite having increasingly more standards of evidence to ensure the safety of patients and work teams [10], dental professionals feel fear and anxiety while working in their respective fields due to the impacts of the pandemic [12,13].

The use of adequate protection is essential to avoid the transmission of SARS-CoV-2. The effectiveness of prevention measures is still limited. In the dental field, a reassessment of risks to which both dentists and dental hygienists are subjected, as well as the rest of the clinic staff, should be performed, formulating feasible protection measures and promoting their implementation in dental clinics. Public health institutions and the Spanish Dentists Association have kept members informed about protection measures in dental professional teams, preparing protocols, reports, and infographics of interest that have been updated according to scientific evidence at all times [14].

The following of clinical practice guidelines for the prevention of respiratory diseases on the part of health personnel has been highly variable [15]. Factors such as the way in which the recommendations are disseminated, training of health and clinical management personnel, accessibility to PPE for the entire team, together with the confidence and motivation to provide adequate patient care constitute the determining factors for the degree of workers’ compliance with the guidelines for prevention and control of respiratory infection [15,16,17]. After the success of the “Dental Action Plan for the Post-Epidemic COVID-19 Period”, which had more than 10,000 Spanish dentists enrolled [18], the General Council of Dentists and the Spanish Dental Foundation launched a free online training programme at the end of April 2020, aimed at dental hygienists and clinical assistants so that they could work with the best protection guaranties.

The analysis of COVID-19 cases notified to the National Epidemiological Surveillance Network (RENAVE) from 11 May 2020, after the entry into force of the new Surveillance and Control Strategy in the transition phase of the pandemic, using the SiViES platform indicated that on 17 February 2021, health or social health personnel represented 4.4% of COVID-19 cases in Spain, with greater impacts on women (6.6%) in comparison to men (2.0%). Women represented 78% of the health or social health personnel with COVID-19. With respect to the characteristics of the environment of possible exposure, 2% of cases were attributed to health centres [19]. This report reflects the variability of the percentage distribution of COVID-19 cases affecting health professionals according to the reports provided by the autonomous communities [19].

From a global perspective, the estimated data on COVID-19 infections and deaths in healthcare workers during the early phases of the pandemic showed that while deaths were mainly in men (70.8%) and doctors (51.4%), infections were mainly in women (71.6%) and nurses (38.6%), suggesting that general practitioners and mental health nurses were the highest risk factors for death. Furthermore, the estimated global data from early phases revealed that Europe had the highest absolute numbers of reported infections and deaths, with the Eastern Mediterranean region having the highest numbers [20]. Indeed, globally reviewed data also pointed to Italy as one of the worst-affected countries worldwide from the beginning of the COVID-19 epidemic, showing that 11.9% of all confirmed cases were healthcare workers [21]. Recent data from 37 countries revealed that nearly 300,000 healthcare workers were infected with COVID-19 by 15 August 2020, with over 2500 deaths [22]. Among dental hygienists, there is still little published information about the pandemic’s influence on their profession worldwide. In the United States of America, the estimated prevalence rate of COVID-19 seems to be low (3.1%) [23] but higher than rates found in international surveys of dental hygienists [24,25]. Despite the enhanced infection control efforts reported in 99.1% of dental practices, it has been suggested that there is a need for further support for dental hygienists’ use of PPE [23].

The objective of the present study was to describe the employment situation of dental hygienists caused by the COVID-19 pandemic in Spain during the de-escalation phase (June 2020) in order to analyse the standards of knowledge and the use of PPE, as well as the limitations of dental activity or changes in the forms of service provision, both in public and private health services.

## 2. Material and Methods

### 2.1. Sample and Procedure

The study was approved by the Bioethics Committee of Murcia University (Reference Number: 2842/2020).

The study population consisted of registered dental hygienists from private practices and the Spanish National Health System. A retrospective questionnaire was administered from 28 May 2020 to 12 June 2020 (between this AND that). The survey was designed using the Survey Platform of the University of Murcia (available online: https://encuestas.um.es/encuestas/inicio.publico.logincas.genParticipants (accessed on 28 June 2020)). The questionnaire was sent via WhatsApp, Facebook, and Instagram; it was anonymous and all participants were informed that completion implied consent to participate in the study. The survey was administered using the ENCUESTAS Platform (University of Murcia) Available online: https://encuestas.um.es/encuestas/covid19desescalada.cc (accessed on 28 June 2020).

A pilot questionnaire was tested on 45 hygienists from 12 Spanish Autonomous Communities (political and geographical regions) and 10 Spanish oral epidemiologists, most of whom were university professors, to ensure the questionnaire questions had been correctly designed, were easily understandable, and did not require a prolonged response time.

### 2.2. Study Instrument

The questionnaire was divided into two main sections. The first section covered sociodemographic characteristics.

### 2.3. Variables Studied

The studied variables are listed below:

- Sex: Male/Female;
- Age (mean age);
- Years of work experience (mean age);
- The Autonomous Community where you work: 16 Spanish Autonomous Communities;
- Environment of work:
  ○ Rural area (<20,000 inhabitants);
  ○ Semi-urban area (20,000–100,000 inhabitants);
  ○ Urban area (>100,000 inhabitants);
- Field of work:
  ○ Public;
  ○ Private.

The second section consisted of questions related to the occupational situation, symptoms related to COVID-19, biological protection and the use of approved PPE, the risk of contagion, and symptoms and general health status during pandemic period. Some questions used multiple-choice answers.

- Dental care of patients:
  ○ I continue to work treating scheduled patients;
  ○ I am still working in my usual position, with no aerosol activities;
  ○ I am still working in my usual position, treating only dental emergencies;
  ○ I am located at home attending only emergencies;
  ○ I have stopped working by own decision;
  ○ I have stopped working due to dismissal;
  ○ I have stopped working completely.
- In case you have returned to work, what measures have you included for patients? (multiple choice)
  ○ Quote spacing;
  ○ Hand washing with hydroalcoholic gel;
  ○ Mandatory mask;
  ○ Patient cap;
  ○ Patient shoe covers;
  ○ Mouthwash for the patient before treatment.
- In case you have returned to work, what measures have you included in the dental clinic? (multiple choice)
  ○ Disinfection for shoes at the entrance;
  ○ Partition screen in reception;
  ○ Safety distance signalling;
  ○ Readaptation of spaces in waiting room;
  ○ HEPA filters.
- Do you have PPE for staff at the dental office?
  ○ Yes, for everyone;
  ○ Yes, but only for the dentist, not for the whole team;
  ○ We do not have PPE.
- What type of masks do you use mainly?
  ○ Surgical;
  ○ FFP2 always;
  ○ FPP2 + surgical;
  ○ FPP2 and FFP3 occasionally;
  ○ FPP3 always;
  ○ FPP3 + surgical.
- Do you use another type of PPE? (multiple choice)
  ○ Cup;
  ○ Protective glasses;
  ○ Facial screen;
  ○ Disposable gown;
  ○ Waterproof suit on top of regular work clothes;
  ○ Shoe covers;
  ○ Double globes.
- Information on protective measures necessary to prevent professional infection that you have introduced in your clinic has come from:
  ○ Health service management;
  ○ Clinic management;
  ○ The Council of Dentist/Professional Colleges;
  ○ From my personal search on web and social networks;
  ○ I have not received any information.
- Have you complied with official recommendations to prevent infections?
  ○ Yes, always;
  ○ Almost always;
  ○ Sometimes;
  ○ Almost never;
  ○ Never.
- I identify more with:
  ○ Return to work without restrictions;
  ○ Return to work progressively, limiting aerosols;
  ○ Only attend emergencies;
  ○ Dental activity should not have been started.
- Symptoms compatible with COVID-19 infection (multiple choice):
  ○ Cough;
  ○ Fever (more than 37.5 °C);
  ○ Vomiting;
  ○ Diarrhoea;
  ○ Sore throat;
  ○ Headache;
  ○ Muscle pain;
  ○ Unexplained tiredness;
  ○ Dyspnoea;
  ○ Alterations in smell and taste;
  ○ Serious lung problems;
  ○ I have not had any symptoms.
- Duration of the symptoms:
  ○ 1–3 days;
  ○ 4–7 days;
  ○ 8–13 days;
  ○ >14 days.
- Have you been in self-isolation for two weeks for COVID-19?
  ○ Yes, because of a confirmed case;
  ○ Yes, because of a suspected case;
  ○ Yes, because of a close contact;
  ○ I have not been in isolation.
- Have you been in isolation for 2 weeks due to COVID-19?
  ○ Yes, because of a confined case;
  ○ Yes, because of a suspected case;
  ○ Yes, because a close contact;
  ○ I have not been in solitary isolation.
- Have you ever taken a SARS-CoV-2 test?
  ○ No, I do not want to do it;
  ○ No, but I am waiting to do it;
  ○ Yes, positive PCR;
  ○ Yes, negative PCR;
  ○ Yes, positive quick test;
  ○ Yes, negative quick test.
- Have you ever had any of these symptoms? (Table 1)

- In case of migraine, you associate it with:
  ○ Stress due to pandemic situation;
  ○ Personnel problems;
  ○ Caused by COVID-19;
  ○ Use of mask FFP2 or FFP3.
- For how long do you use FFP2/FFP3 mask continuously?
  ○ I take off every hour and rest for a few minutes;
  ○ I wear it for 3 h continuously;
  ○ I wear it for more than 3 h without taking it off.

### 2.4. Data Analysis

Data were processed and analysed using the R statistical software. To identify the association level in the categorical variables regarding the sociodemographic variables, Pearson’s chi-square test was used in contrasts where the required assumptions were met, while Fisher’s exact test was used when they were not (data independence and expected values of frequencies greater than 5). To identify statistically significant differences in ordinal variables regarding sociodemographic variables, the Kruskal–Wallis test was used (*p*-value < 0.05 and level of significance 0.05), as this test is the most robust for this type of data.

A cross-sectional study was performed, which complied with the STROBE norms on cross-sectional studies (available online: http://www.strobe-statement.org (accessed on 17 April 2020)).

## 3. Results

### 3.1. Descriptive Analysis

A total of 517 responses were obtained from dental hygienists, of whom 5.8% were male and 94.2% female, with a mean age of 40.43. The mean for years of professional experience was 14.95. The distribution of the sample by geographical region is shown in Table 1.

In this sample, 49.3% of respondents were active in urban areas, 34.1% in semi-urban areas, and 16.6% in rural areas. Regarding the type of activity, 78.5% of respondents worked in private practices and 21.5% in the public health system (Table 2).

### 3.2. Inferencial Analysis

During the de-escalation period between 28 May and 12 June 2020, 51.6% of the respondents working in the private sector had re-entered the labour force, performing all types of dental activities, compared to 4.1% in the public sector (*p* < 0.005). Among those who re-entered but did not perform aerosol-generating activities, 11.61% did so in private practices compared to 8.9% in public practices (*p* < 0.005). It was found that 7.5% of the hygienists in private practices stopped working due to being laid off or furloughed or due to closure (*p* < 0.005) (Table 3). Furthermore, 4.6% of the professionals working in the private sector only performed emergency dentistry, a value slightly lower than for professionals in the public sector (6.6%) (*p* < 0.005).

As for the new measures adopted by patients, these included hand washing with hydroalcoholic gel (15.9% in private practices and 4.5% in public practices), mouthwash rinsing prior to clinical work (15.6%, with significant differences compared to public practices; *p* < 0.05), and spacing of patient appointments in 14% of private practices compared to 4.5% in public practices (*p* < 0.005). The use of patient caps (9%) and shoe covers (10.4%) in private practices also exceeded the values in public practices (*p* < 0.05). HEPA filters for air conditioning were incorporated in 4.9% of dental clinics, a value much higher than that found in public practices (*p* < 0.005) (Table 3).

Regarding PPE, filtering phase piece masks (FFP2) together with surgical masks were used in 76% of consultations, although with a large difference between private sector (60.5%) and public sector usage (15.5%), along with caps 19.5% and face shields 18.5%. The use of double gloves in private practice (11.7%) compared to public practice (1.4%; *p* < 0.05) also differed during the pandemic.

Information on the protective measures necessary to avoid professional infection was provided by the Council of Dentists/Professional Colleges (39.4%), with private practices always prevailing over public practices (*p* < 0.005) and those in charge of the practices (31.1%). Of those interviewed, 86.2% stated that the official recommendations should be followed to avoid contagion and identified with a gradual return to work by limiting the use of aerosols 68.3%; however, private dental hygienists identified more with a return to work without restrictions (14.5%) compared to those working in the public sector (1.2%) (*p* < 0.005) (Table 3).

Approximately 50.5% of dental hygienists interviewed had no symptoms compatible with COVID-19 infection, with a difference between private and public practices (*p* < 0.05); the most frequent symptoms included headache (46.7%), sore throat (35.1%), and muscle pain (27.7%). A total of 2.5% of hygienists surveyed were in isolation for two weeks as a confirmed COVID-19 case.

Here, 17.2% of hygienists had taken PCR tests and 18.9% had taken rapid tests, of which only 1% of PCR tests and 0.9% of rapid tests were COVID-19-positive. The percentages of diagnostic tests performed according to work setting showed that 78% (*n* = 87) of hygienists in the public health system and 74.3% (*n* = 105) hygienists in private practices had taken tests (*p* < 0.05) (Table 4).

Among the pathologies that have emerged after the pandemic, migraines (26.54%) together with insomnia (39.29%) (*p* < 0.05) and irritability (37.3%) (*p* < 0.05) stood out. Some respondents even reported post-pandemic depression (16.6%), which was much higher among professionals in private practices (Table 4). No association was found between suffering from headache or migraine and worsening effects after the pandemic among professionals in public or private practice (*p* > 0.05), despite the increases in continuous use of FFP2/FFP3 masks for more than 3 h (53.2%), less than 3 h (18.2%), and hourly (28.7%) (Table 5).

## 4. Discussion

After the first phase of infections in Spain, when very deficient means of protection were available [24,26], health professionals experimented with different strategies to face return to work and control of infection in dental clinics, ensuring the safety of patients and professionals. To our knowledge, no data have been published on the situation of the dental profession after the first de-escalation phase of the COVID-19 pandemic in Spain.

The main limitations of the present study were the random sampling and sample size (*n* = 517), since in Spain there is no official registry of dental hygienists and we could not calculate the total number of these oral health workers; due to this, we were also unable to estimate the actual response rate for our questionnaire. In our study, women were predominant (*n* = 487, 94.2%), which is consistent with the fact that the number of female oral health professionals is greater than that for males [27]. The wide geographical distribution and the representation of the public and private healthcare settings minimised the possible bias in the resulting data; however, these results cannot be extrapolated to the country as a whole. This methodology (i.e., via social networks) [24,26,28] was chosen because it allows faster and broader access to the intended populations.

Some of the main concerns for oral health professionals after the coronavirus outbreak were the economic repercussions that the closure or reduction of the number of patients in dental centres caused [29,30], the increases in costs, and the difficulty in acquiring PPE, in addition to new investments in resources or infrastructures [30,31]. During these critical months, the Government of Spain approved a Royal Decree, called ‘ERTE’, for the relaxation of conditions in private companies, favouring temporary layoffs with suspensions of contracts or reductions of working hours, avoiding the definitive closure of dental clinics or collective dismissals [29]. The design of the present study prevented us from affirming whether the 8.1% of dental hygienists who were still not working had accepted ERTE in order to avoid dismissals or closure of the clinics, or whether an employment regulation, called ‘ERE’, had been implemented; however, the analysis indicated that 2.8% had definitively stopped working due to the economic situation or their own decisions. Some studies have considered that governments should help workers of financially supported dental centres in their adaptation of investments made to promote safe clinical practices in their dental clinics during the COVID-19 pandemic [31].

Stress among dental hygienists and other professionals working in dental clinics is a common factor associated with the potential risk of infection from aerosol-generating procedures (AGP), which are performed during their daily activities, along with concerns related to infection control [30,32,33,34]. From a professional point of view, the results show that the return to work should have been carried out progressively and while limiting the production of aerosols, although it 55.7% of the dental hygienists interviewed during the de-escalation period returned to work as usual, performing all types of treatments without taking into account whether or not aerosols were generated during the procedure. Only 20.5% have admitted that they do not perform AGP; public health care workers followed this recommendation the most. Clinical practice strategies should emphasise minimizing AGP as much as possible; minimally invasive and atraumatic dental treatment alternatives should be chosen [25,35]. The dental hygienist’s competence in periodontal treatment and maintenance should consider implementing safe manual plaque removal techniques instead of mechanical interventions, which are considered high AGP [33]. Preventive dental services have been one of the main services with the most delays during the pandemic, and this situation may influence the prognosis of oral health conditions, so other viable alternatives such as telemedicine should be explored to make up for these deficiencies [36].

The reintroduction of scheduled activity in dental clinics due to the COVID-19 pandemic in the de-escalation phase should prioritise the safety of patients and professionals at the different stages of the care process, from patient selection and preparation to home care and clinical circuit organisation [37]. Among the preventive measures adopted by patients, the results of this study on Spanish dental hygienists coincide with the measures assessed in other previous studies (e.g., [25]), such as hand washing with hydroalcoholic gels, mouthwash rinsing prior to treatment, or spacing of appointments to avoid saturation in waiting rooms [27]. According to the data presented from the Spanish hygienists survey, appointment time management is the main measure taken in the oral health units of the public health service. According to Moffat et al. in their study on the identification of patients’ perceptions of risk susceptibility and attitudes towards COVID-19 [37], professionals and public institutions should adopt additional information measures aimed at patients to address the conditions, events, and fears that influence the delay of their visits or the low demand for dental care in this context, in order to ensure the safe reopening of clinics and to regain patient confidence [38,39].

The measures incorporated in dental practices for their adaptation, according to this study, have not required large investments in HEPA filter suction systems (5.3%), nor in other air purification systems (7.8%), which evacuate the air from an area by dissipating the atmosphere. The vacuum system type may influence the risk of aerosol transmission of SARS-Cov-2 [40], but it should be considered as a complementary infection control measure considering the functionality and limitations of HEPA filter systems [41]. Recent studies have confirmed that if there is no availability for mechanical ventilation, natural ventilation may not be recommended during activities of competence, such as ultrasonic scaling or when using airborne spreading devices, which under standard conditions seem to release high levels of particulate matter that can act as vectors of infectious agents and contaminate patients and dental practitioners [42]. As in other studies [30,34], physical barriers such as partitions, readaptation of waiting rooms, and signage of safety distances have been the main changes implemented in Spanish public and private dental practices.

The majority of hygienists surveyed use FFP2 or higher-filtration FFP3 masks, while only 10.2% admitted not using them and working only with surgical masks. If we compare these data with the surveys carried out during March and April 2020 in Spain, we can see that in the de-escalation phase the availability of FFP2 or FFP3 masks has improved among dental hygienists and the rest of the dental team, dissipating the gap in access to this PPE that was evident between professionals in the private sector and dental hygienists [35]. The usage rates of other PPE such as face shields, goggles, double gloves, caps, lab coats or waterproof suits, and shoe covers were similar to those reported in the surveys of the International Federation of Dental Hygienists (IFDH) [24].

The management of the training given to dental hygienists and auxiliary staff for infection prevention and control has not been widely evaluated, although it was essential in limiting the spread of infection, involving a whole-team effort [43,44]. In Spain, the online training for dentists named the “Dental Action Plan for the Post-Epidemic COVID-19 Period” promoted by the General Council of Dentists and the Spanish Dental Foundation reached approximately half of the registered Spanish dentists [18]. The readaptation of the format for hygienists and auxiliary staff for this training at the end of April 2020 reached 39.4% of surveyed dental hygienists who confirmed that they received information from this organisation, although 31.1% stated that this had been provided by the practice managers. The study by Shahin et al. [43] conducted among dentists, dental assistants and hygienists, and dental undergraduates revealed certain deficiencies in training, as 93.5% of dental hygienists did not believe that rubber dam isolation was a SARS-CoV-2 prevention measure [43]. In addition, erroneous beliefs regarding bacterial antiseptics and their effectiveness with SARS-CoV-2 were reiterated [44]. This implies a knowledge gap regarding preventive measures, which have not been questioned among Spanish hygienists and should be emphasised in order to minimise the risk of infection. Regarding the adherence to official recommendations to prevent infection, 82.6% of dental hygienists in both the public and private sectors indicated their compliance. Estrich et al., when analysing this aspect in dental hygienists in the United States and using the recommendations of the Centres for Disease Control and Prevention (CDC) as a reference, found that 55% followed these recommendations and stated that the rate was higher among professionals with more years of experience [23]. According to other surveys, confidence levels in performing dental hygienist activities safely to avoid SARS-CoV-2 infection showed significant differences depending on professional experience [39,43,44].

The lack of diagnostic tests during the first months of the pandemic may explain the fact that 38% of respondents (*n* = 201) were waiting for a diagnostic test (either PCR or rapid antigen test). Considering that 87.4% of hygienists had returned to clinical activity at that time, 26.30% of the sample did not follow the health surveillance instructions for workers, not only to protect their health, but also to prevent transmission to patients or between teams of professionals [25]; however, the diagnostic capacity in dental hygienists has increased compared to the data from the first survey in early April 2020, which mentioned that only 1.1% of hygienists and auxiliary staff had been tested. The study performed by Estrich et al. in the USA showed that approximately one-third of their sample (35.4%) had been tested at least once with some kind of test [23], which could be considered worse compared to Spanish hygienists, as the American survey was carried out in October 2020. In terms of respondents’ symptomatology, the values were similar to the studies conducted in Italy, where 81.61% of respondents had no symptoms. The descriptions of compatible symptoms were similar to those described in a study conducted in the USA, where headache (32.2%), muscle pain (17.7), and fatigue (17.3%) prevailed [23].

The general health status of dental hygienists during the pandemic is essential to the effectiveness and quality of their performance. This study has found a significant association between the increase in sleep disorders or insomnia among hygienists coinciding with the post-pandemic period (39.3%, *n* = 203). A similar study carried out on Polish dentists revealed that sleep parameters, both in terms of quality and in number of hours, decreased during the pandemic, in addition to other significant symptoms such as headache, back, and neck pain [45]. Irritability, migraines, fear, and depression were also measured but with lower levels of relevance, although according to 34.6% of the respondents these factors were associated with the stress of the pandemic situation, while to a lesser degree 17.1% of respondents these factors were associated with the use of FFP2/FFP3 masks, which more than half of the hygienists use for more than 3 h continuously. The study by Shacham et al. among dentists and dental hygienists [30] and the meta-analysis by Maqbali et al. among nurses [46] confirmed that stress, fear of contracting the disease, and work overload are the main triggers of anxiety, depression, and sleep disorders. The subjective burden and psychological distress among professionals in the dental sector differ between countries according to social, cultural, and environmental factors [47]. It is recommended that psychological support services, workshops, or training be provided to reduce the impact among dental professionals [48,49].

We have previously discussed the limitations of not knowing the exact number of dental hygienists working in Spain. Furthermore, another limitation of this study is that although we used an analogous questionnaire with dentists in a previous work [39], it had not been fully validated in dental hygienists before; thus, another possible weakness of the present work could be the caution required when generalizing the results to similar studies carried out in other countries in the present or in the future.

## 5. Conclusions

Dental hygienists’ return to work during the de-escalation phase in Spain has involved different strategies aimed at infection control and ensuring the safety of patients and the rest of the dental team. The activities of dental hygienists in private centres have been carried out without progressively limiting aerosol-generating procedures. The lack of implementation of care provision based on telemedicine in continuing the dental educational process in terms of the delivery of didactic components, clinical training, and patient care or minimal intervention techniques that do not generate aerosols may have influenced the results. In contrast, dental hygienists in the public sector have limited these interventions by avoiding the generation of aerosols during this period; however, the availability of personal protective equipment, the rehabilitation of clinical infrastructure, and patient management should ensure confidence and safety in dental care. This group’s health surveillance should be improved, both to avoid the transmission of COVID-19 to patients or between teams of professionals and to prevent psychological and sleep disorders due to the stress generated during the pandemic, which affect their work efficiency and quality.

## Figures and Tables

**Table 1:** Possible Symptoms (migraine headache, insomnia, irritability, depression and fear) suffered pre-pandemic and post-pandemic.

Symptoms	Pre-Pandemic		Post-Pandemic	
	Yes	No	Yes	No
Migraine headache				
Insomnia				
Irritability				
Depression				
Fear				

**Table 2:** Geographical distribution and sociodemographic characteristics of the sample regarding field of work.

Field of Work	Public *n* (%)	Private *n* (%)	Total *n* (%)
**Participants**	111 (21.5)	406 (78.5)	517 (100)
—Male	8 (26.6)	22 (73.3)	30 (5.8)
—Female	103 (21.1)	384 (78.9)	487 (94.2)
—Rural	16 (18.6)	70 (81.4)	86 (16.6)
—Semi-urban	39 (22.2)	137 (77.8)	176 (34.1)
—Urban	56 (22)	199 (78)	255 (549.3)
**Spanish Autonomous Communities**			
Andalucía	15 (23.1)	50 (76,9)	65 (12.57)
Aragón	13 (27.1)	35 (72.9)	48 (9.3)
Asturias	2 (22,2)	7 (77,8)	9 (1.7)
Basque Country	0	11 (100)	11 (2.1)
Cantabria	1 (25)	3 (75)	4(0.7)
Castilla Leon	9 (26.5)	25 (73.5)	34 (6.5)
Castilla-La Mancha	11 (39.3)	17 (60.7)	28 (5.1)
Cataluña	1 (4)	24 (96)	25 (4.8)
Extremadura	7 (58.3)	5 (41.6)	12 (2,32)
Galicia	2 (11.8)	15 (88.2)	17 (3.3)
Islas Baleares	11 (45.8)	13 (54.2)	24(4.6)
Islas Canarias	5 (45.5)	6 (54.5)	11 (2.1)
La Rioja	0	1 (100)	1 (0.2)
Madrid	13 (4.9)	48 (95.1)	61 (11.8)
Murcia	3 (21.3)	48 (78.7)	51 (9.9)
Navarra	0	17 (100)	17 (3.3)
Valencia	18 (18.2)	81 (81.8)	99 (19.1)
			**Mean (max. value–** **min. value)**
Age			40.43 (65-19)
Years of Practice			14.95 (58-0)

**Table 3:** Dental care and PPE used by hygienists after first COVID-19 peak of the pandemic.

Questions	Public *n* (%)	Private *n* (%)	*p*-Value
**Dental care on patients**			
—I continue to work treating scheduled patients.	21 (4.1)	267 (51.6)	<0.005 *
—I continue to work in my usual position, with no aerosol activities.	46 (8.9)	60 (11.61)	<0.005 *
—I continue to work in my usual position, treating only dental emergencies.	34 (6.6)	24 (4.6)	<0.005 *
—I am located at home attending only emergencies.	1 (0.2)	8 (1.5)	>0.05
—I have stopped working by own decision.	4 (0.8)	6 (1.2)	>0.05
—I have stopped working due to dismissal.	3 (0.6)	39 (7.5)	<0.05 *
—I have stopped working completely.	2 (0.4)	2 (0.4)	>0.05
			<0.001 *
**If you have returned to work, what measures have you included for patients?**			
—Quote spacing	98 (4.5)	304 (14.0)	<0.005
—Hand washing with hydroalcoholic gel	95 (4.4)	345 (15.9)	0.992
—Mandatory mask	93 (4.3)	320 (14.8)	0.306
—Patient cap	41 (1.9)	196 (9.0)	<0.05
—Patient shoe covers	28 (1.3)	225 (10.4)	<0.005
—Mouthwash for the patient before treatment	83 (3.8)	339 (15.6)	<0.05
			<0.001 *
**New protection measures included in dental surgery**			
—Disinfection for shoes at the entrance	42 (3.1)	198 (14.6)	0.05 *
—Partition screen in reception	34 (2.5)	288 (21.2)	<0.001 *
—Safety distance signalling	47 (3.5)	158 (11.6)	0.586
—Readaptation of spaces in waiting room	79 (5.8)	355 (24.7)	<0.05 *
—HEPA filters	5 (0.4)	66 (4.9)	<0.005 *
—Others air purification systems	10 (0.7)	96 (7.1)	<0.005 *
			<0.001 *
**What type of masks do you use mainly?**			
—Surgical	13 (2.5)	40 (7.7)	0.692
—FFP2 always	13 (2.5)	43 (8.3)	0.869
—FFP2 + surgical	80 (15.5)	313 (60.5)	0.330
—FFP2 and FFP3 occasionally	1 (0.19)	3 (0.6)	>0.05
—FFP3 always	0	1 (0.19)	>0.05
—FFP3 + surgical	4 (0.77)	6 (1.16)	>.05
			>0.05 **
**Do you use any other type of PPE?**			
—Cup	98 (4.0)	378 (15.5)	0.142
—Protective glasses	73 (3.1)	280 (11.5)	0.868
—Facial screen	98 (4.0)	353 (14.5)	0.829
—Disposable gown	72 (2.9)	244 (10%)	0.422
—Waterproof suit on top of regular work clothes	52 (2.13)	(221 (9.1)	0.189
—Shoe covers	34 (1.39)	158 (6.5)	0.136
—Double globes	92 (1.4)	285 (11.7)	<0.05 *
			>0.05 **
**Information on protective measures necessary to prevent professional infection that you have introduced in your clinic has come from:**			
—Health service management.	52 (5.9)	48 (5.5)	<0.005 *
—Clinic management.	44 (5.1)	227 (26.0)	<0.005 *
—The Council of Dentists/Professional Colleges.	53 (6.1)	290 (33.3)	<0.005 *
—From my personal search on websites or social networks.	36 (4.1)	109 (12.5)	0.297
—I have not received any information.	7 (0.8)	6 (0.7)	<0.005 *
			<0.05 **
**Have you complied with official recommendations to prevent infections?**			
—Yes, always	97 (18.8)	330 (63.8)	
—Almost always	12 (2.3)	60 (11.6)	
—Sometimes	0	11 (2.1)	
—Almost never	1 (1.2)	4 (0.7)	
—Never	1 (0.2)	1 (0.2)	
			>0.05 ***
**I identify more with:**			
—Return to work without restrictions	6 (1.2)	75 (14.5)	>0.005
—Return to work progressively, limiting aerosols	77 (14.9)	276 (53.4)	0.870
—Only attend emergencies	19 (3.7)	27 (5.22)	>0.005
—Dental activity should not have been started	9 (1.7)	28 (5.4)	0.817
			>0.001 *

Note: * X^2^ Pearson test; ** Fisher test; *** Mann-Whitney U test.

**Table 4:** Symptoms and professional isolation due to COVID-19 among dental hygienists.

Questions	Public *n* (%)	Private *n* (%)	*p*-Value
**Have you had symptoms compatible with the COVID-19 infection during the month of February, March, or April?**			
Cough	12 (1.5)	21 (2.7)	0.053
Fever (>37.5 °C)	6 (0.8)	18 (2.3)	0.859
Vomiting	0	2 (0.2)	>0.05
Diarrhea	13 (1.7)	26 (3.3)	0.094
Sore throat	14 (1.8)	52 (6.7)	>0.05
Headache	22 (2.8)	66 (8.5)	0.458
Muscle pain	13 (1.7)	34 (4.4)	0.369
Unexplained tiredness	12 (1.6)	33 (4.2)	0.484
Shortness of breath	5 (0.6)	9 (1.2)	>0.05
Loss of taste and smell	8 (1.0)	16 (2.1)	0.232
Difficulty breathing	1 (0.1)	1(0.1)	>0.05
No symptoms	75 (9.7)	317 (40.8)	<0.05 *
			>0.05 *
**If you have had symptoms, please indicate the duration**			
1–3 days	7 (5.7)	34 (27.87)	0.093
4–7 days	10 (8.2)	17 (13.9)	>0.05
8–13 days	9 (7.4)	22 (18.0)	>0.05
>14 days	8 (6.6)	15 (12.3)	0.574
			>0.05 *
**Have you been in solitary isolation for two weeks due to COVID-19?**			
Yes, because of a confirmed case	6 (1.2)	7 (1.3)	<0.05 **
Yes, because of a suspected case	7 (1.3)	24 (4.6)	>0.05
Yes, because of close contact	7 (1.3)	1 (0.2)	<0.05 **
I have not been in solitary isolation	91 (17.6)	374 (72.34)	<0.05 *
			<0.05 **
**Have you ever taken any SARS Cov-2 test?**			
Not, I do not want do it	5 (1)	119 (23.1)	<0.05 *
No, but I am waiting to do it	19 (3.7)	182 (35.2)	<0.05 *
Yes, positive PCR	5 (1)	0	<0.05 **
Yes, negative PCR	39 (7.5)	45 (8.7)	<0.05 *
Yes, positive quick test	1 (0.2)	4(0.7)	<0.05 **
Yes, negative quick test	42(8.1)	56 (10.8)	<0.05 *
			<0.05 **

Note: * X^2^ Pearson test; ** Fisher test.

**Table 5:** General health during the pandemic period.

Questions	Public *n* (%)	Private *n* (%)	*p*-Value
**Have you ever had any of these symptoms?**			
—Migraine headache No pre-/No post-pandemic	44 (8.5)	171 (33.1)	0.718
—Migraine headache No pre-/yes post-pandemic	37 (7.2)	100 (19.34)	0.085
—Migraine headache Yes pre-/No post-pandemic	23 (4.4)	104 (20.12)	0.348
—Migraine headache Yes pre-/Yes post-pandemic	7 (1.3)	31 (6)	0.786
			>0.05
—Insomnia No Pre-/No Post-pandemic	41 (7.9)	201 (38.9)	<0.05 *
—Insomnia No pre-/Yes Post-pandemic	51(9.89)	152 (29.4)	<0.05
—Insomnia Yes pre-/No Post-pandemic	18 (3.5)	40 (7.7)	0.08
—Insomnia Yes pre-/Yes Post-pandemic	1 (0.2)	13 (2.5)	>0.05
			<0.05 **
—Irritability No Pre-/No Post-pandemic	54 (10.4)	222 (42.94)	0.307
—Irritability No Pre-/Yes Post-pandemic	49 (9.5)	144 (27.8)	0.117
—Irritability Yes pre-/No Post-pandemic	7 (1.3)	25 (4.8)	<0.05 *
—Irritability Yes pre-/Yes Post-pandemic	1 (0.2)	15 (2.9)	<0.05 **
			<0.05 **
—Depression No Pre-/No Post-pandemic	82 (15.8)	313 (60.5)	0.560
—Depression No Pre-/Yes Post-pandemic	20 (3.9)	66 (12.7)	0.765
—Depression Yes pre-/Yes Post-pandemic	9 (1.7)	23 (4.4)	0.468
—Depression Yes pre-/Yes Post-pandemic	0	4 (0.7)	<0.05 **
			<0.05 **
—Fear No Pre-/No Post-pandemic	79 (15.3)	268 (51.84)	0.361
—Fear No Pre-/Yes Post-pandemic	5 (1)	27 (5.2)	0.542
—Fear Yes pre-/Yes Post-pandemic	12 (2.3)	52 (10.1)	0.686
—Fear Yes pre-/Yes Post-pandemic	15 (2.9)	59 (11.4)	0.905
			>0.05 *
**In case of headache you associate it with**			
—Stress due to pandemic situation	49 (6.8)	176 (27.8)	0.966
—Personnel problems	15 (2.4)	48 (7.6)	0.749
—Caused by COVID-19	6 (0.9)	13 (2.0)	<0.05
—Use of FFP2 or FFP3 mask	29 (4.6)	79 (12.5)	0.61
			>0.05 **
**How long do you use FFP2/FFP3 mask continuously**			
—I take off every hour and rest for a few minutes	52 (10.1)	96 (18.6)	<0.05 *
—I wear it for 3 h continuously	29 (5.6)	65 (12.6)	<0.05 *
—I wear it for more than 3 h without taking it off	30 (5.8)	245 (47.4)	<0.05 *
			<0.05 *

Note: * X^2^ Pearson test; ** Fisher test.

## Data Availability

The datasets used for the current study are available from the corresponding author upon reasonable request.
